# Tyner’s Cataract Operation

**Published:** 1895-10

**Authors:** 


TYNER'S CATARACT OPERATION.—It will be remembered by readers of current medical literature that the distinguished Austin oculist, Dr. T. J. Tyner, read a paper at the 11th International Medical Congress at Rome, Italy, last year, which created quite a breeze amongst the many eye specialists present. The subject was "*Preliminary Capsulotomy in Cataract Extraction*," a method devised by himself, and which he had been using with much success for some years.

It was a new departure; something they had not heard of, and it at once awakened a lively interest. The paper was translated into French, German, Italian and Spanish, and telegraphed to the leading publications. Dr. Tyner has ever since been receiving letters from physicians in Europe and America, making inquiry as to the *technique* of the operation, etc. To answer these letters has been quite an onerous task, and we suggested to the doctor the expediency of making known through the TEXAS MEDICAL JOURNAL the data these gentlemen were asking for. In compliance with our request, the doctor has given us the following. The doctor says:

DEAR DOCTOR:—This differs from other methods only in making the capsulotomy the primary step in the operation. This is done with an ordinary Bowman's needle, the pupil being dilated with a weak solution of atropia. The needle is introduced at a point about where the knife enters in making the corneal incision; the capsule is opened in its upper peripheral quadrant. Sometimes, if the sac is tough, a vertical opening is made, the two forming the letter "T."

Much care is necessary in this manœuvre, especially in withdrawing the needle, not to lose any of the aqueous. Should this occur in sufficient quantity to cause an appreciable collapse of the globe, the eye should be closed for a few minutes, until the humor reaccumulates.

In making the corneal incision, just as the section is being finished, a little pressure with the flat of the blade causes the edge of the lens to tilt forward; then as the knife emerges, slight counter-pressure with the fixing-forceps below, forces it (the lens) to follow the knife, and glide out with surprising ease. That this is true follows from the fact, the capsule being open, the lens from the pressure, naturally seeks the point of least resistance.

Another advantage in the operation is this: If the suspensory ligament is weak, the lens is easily dislocated with the needle; I have done this in a number of cases, and that, too, without the loss of vitreous. Regarding this accident, I find it less frequent than in other methods. I do not remember having a case of prolapse of the iris, and rarely iritis. In a word, the after-management has been attended with but little trouble. This is probably accounted for by the fact that the traumatism is reduced to the minimum, and the operation more quickly performed.

I use the weakest solution of atropin to dilate the pupil; the reason is obvious. However, in the after-treatment I begin its use earlier, usually the third day. With regard to selection of a needle—choice of the different shaped needles—I find the small Bowman's stop needle best; with it there is less danger of escape of secretions.

The operation was first described in a paper to the *New York Medical Journal*, in 1890, and later in a paper read at the International Medical Congress at Rome, in 1894. Extracts from these papers have appeared in medical journals in this country and also in Europe. I recieved a letter from Naples, a few weeks ago, from a gentleman who had performed this operation in his last five extractions. If I have overlooked any points of interest, please let me know.

The writer has seen Doctor Tyner make the extraction after his method. The operation is simplicity itself and the lens escapes with surprising facility. So simple is it, indeed, that it is "a wonder some one had not thought of it before," as a gentleman writing from Naples said, in a recent letter.

Dr. Tyner studied and practiced general medicine many years before taking the eye as a special practice. By this method, a knowledge of general pathology, and of the effects of medicine *upon the system at large* is acquired; something absolutely necessary to know in order to treat any *organ* intelligently. For some dozen or more years he has limited his practice exclusively to this branch, and the writer can testify to his skill, dexterity and success, born of long experience and thorough knowledge. Tyner's operation will in our opinion supercede all others for the extraction of cataract; the after treatment being greatly simplified and shortened.

